# Suicidal ideation in Chinese adults with schizophrenia: associations with neurocognitive function and empathy

**DOI:** 10.1186/s12888-023-04739-3

**Published:** 2023-05-03

**Authors:** Jie Liu, Ke Zhao, Siyao Zhou, Lan Hong, Yao Xu, Shiyu Sun, Siyu Tong, Liandan Huang, Jiahong Liu, Jieqiong Wang, Na Li, Mengbei Lou, Wei Tang, Zhengmao Cai

**Affiliations:** 1 School of Mental Health, Wenzhou Medical University, Wenzhou, Zhejiang China; 2 Lishui Second People’s Hospital Affiliated to Wenzhou Medical University, Lishui, China; 3 The Affiliated Kangning Hospital of Wenzhou Medical University Zhejiang Provincial Clinical Research Center for Mental Disorder, 325000 Wenzhou, China

**Keywords:** Schizophrenia, Suicide, Personal distress, Nonsocial cognitive, Social cognitive

## Abstract

**Background:**

Suicidal ideation is common among people diagnosed with schizophrenia spectrum disorders and may be related to neurocognitive, social cognitive, and clinical variables. This study aimed to investigate the relationships between suicidal ideation and both neurocognitive function and empathy.

**Methods:**

The sample for this cross-sectional study comprised 301 schizophrenic patients aged 18–44 years. All participants were administered the Beck Scale for Suicide Ideation—Chinese Version (BSI—CV), the Repeatable Battery for the Assessment of Neuropsychological Status (RBANS), the Interpersonal Reactivity Index (IRI), and the Positive and Negative Syndrome Scale (PANSS). The demographic and clinical data of the patients were also collected.

**Results:**

In total, 82 patients reported suicidal ideation. Compared to patients without suicidal ideation, patients with suicidal ideation showed significant differences in the IRI-Personal Distress subscale, PANSS-General Psychopathology symptom scores, and suicide attempts. Moreover, there were moderating effects of neurocognitive function and empathy on the relationship between suicide attempts and suicidal ideation.

**Conclusions:**

These results indicate that the personal distress component of empathy, general psychopathology symptoms and suicide attempts are independent risk factors for suicidal ideation in Chinese adults with schizophrenia. Moreover, neurocognitive function may also be related to suicidal ideation through a moderating relationship. In order to reduce suicidal ideation among patients with schizophrenia, early screening of empathy and neurocognitive function is essential.

## Background

Schizophrenia (SZ) is a serious mental disorder with a high suicide rate [1, 2]. Suicide risk can be assessed based on a multi-stage continuum (suicidal ideation, suicide plan, suicide attempted, and completed suicide [1, 3]). Suicidal ideation, at one end of the continuum, is the basis for the prediction of suicide and suicide prevention among patients with SZ [4]. Meta-analyses have shown that the lifetime risk of suicide for patients with SZ is 4.9% [5], and the lifetime prevalence of suicidal ideation among patients with SZ is 34.5% (95% CI: 28.2 − 40.9%) [1]. Suicide is considered to be one of the leading causes of premature death in patients with SZ [6]. Further, a recent study indicated that the suicide rate among young people with SZ has risen [7].

Many published studies have explored the risk of suicide among patients with SZ, and various risk factors have been found to be associated with suicidality in patients with SZ. These factors include demographic variables (such as male gender, younger age, and higher intelligence quotient), clinical characteristics (such as history of attempted suicide and poor adherence to treatment), and various other variables (such as hopelessness) [7]. In addition, neurocognitive function [8] and social cognition [9, 10] are reported to be risk factors for suicide, and their relationships with suicide have been widely explored in recent years. However, the reported risk factors for suicide among patients with SZ are often inconsistent. Therefore, more research is needed.

Nonsocial (also referred to as neurocognitive) and social cognitive impairments are among the core features of SZ. These impairments have a significant impact on treatment, prognosis, and functional outcomes for patients with SZ [11, 12]. Neurocognition and social cognition are two domains of cognition; neurocognition is a basic function of the central nervous system. To date, studies of patients with SZ have identified various neurocognitive deficits, including significant deficits in attention, executive function, learning, information processing, and memory [13]. However, findings on the association between suicide risk and cognitive impairment in SZ have been inconsistent. Some studies [14–16] have reported that impaired neurocognition in patients with SZ is a risk factor for suicide, while others have reported a positive relationship between suicidal risk and improved cognitive function [17–19], or no association between the two variables [20, 21]. Neurocognitive decline is often associated with aging [22], so the current study focused on young adults aged 18–44 years.

The relationship between social cognition and suicidal ideation has also been of great interest to researchers. Social cognition refers to a range of mental processes based on social interactions, including the perception, interpretation, and reaction to the intentions, personalities, and behaviors of others [23]. In particular, the relationship between interpersonal dysfunction and suicide is receiving increasing attention [24, 25]. The interpersonal theory of suicide suggests that frustrated attributions, perceived burdens, and the acquisition of suicidal competence lead to suicide [26]. Perceptions of failure and pitfalls (encompassing the perceptions of failure and no way forward or escape) have been found to be associated with suicidal ideation in SZ [27]. In other studies, lower scores on the Mayer-Salovey-Caruso Emotional Intelligence Test (MSCEIT) [28], lower scores on the false belief task [29], and negative attribution bias and reactivity to more negative stimuli [9] were all associated with suicidal ideation in SZ patients. However, few studies have assessed the interpersonal aspects of social cognition related to suicidal ideation in SZ.

Empathy is an important element of social cognition and involves the integration of several social processes [30]. Empathy is the ability to understand and respond to the emotional experiences of others [31, 32]. It is a form of interpersonal support and plays an important role in building positive relationships and promoting cooperative behavior [33, 34]. Empathy includes both other-oriented empathy and self-oriented empathy [35]. Other-oriented empathy is the ability to experience and understand the feelings of others; it induces altruistic motivation to help others while distancing oneself from the situation. Other-oriented empathy can be measured with the Perspective Taking (PT) and Empathic Concern (EC) subscales of the Interpersonal Reactivity Index (IRI). Ego-oriented empathy is when an individual lacks a buffering distance and can become overwhelmed by exposure to others' distress. This can be measured by the Personal Distress (PD) and Fantasy (FS) subscales of the IRI. With self-direction, feelings of personal distress may evoke an egocentric motivation (to keep the individual in psychological distress) to alleviate the discomfort of exposing oneself to the distress of others [36, 37]. Some studies suggest that a decreased ability to recognize certain social emotions can impair one’s ability to interact socially, which may increase the risk of suicide [38]. In addition, the perceived sense of burden noted in interpersonal theories of suicide is associated with suicide risk, and the nature of this belief requires consideration of others, which may be associated with higher levels of empathy [26, 37]. While empathy is a core topic in social cognitive neuroscience research [39], it has received less attention in studies of patients with SZ. The available studies have not reached a consensus conclusion regarding the relationship between empathy and suicide in patients with SZ [37, 38]. In addition, to date, no published studies have examined the relationships between suicidal ideation and the two domains of cognition in schizophrenia patients. This study addresses this gap by exploring the relationships between suicidal ideation and both neurocognition and empathy.

Therefore, in this study, a cross-sectional design was employed to explore the factors associated with suicidal ideation in Chinese adults with SZ. This study focused on the relationships between suicidal ideation and both empathy and neurocognition. It was hypothesized that (1) certain demographic and clinical variables would be risk factors for suicidal ideation; (2) there would be correlations between suicidal ideation and both neurocognitive function and empathy in SZ patients.

## Methods

### Participants

A total of 301 inpatients with SZ (within three months of admission and within the acute or subacute stage of the illness) who were hospitalized at the Affiliated Kangning Hospital of Wenzhou Medical University between December 2018 and December 2019 participated in this study. All patients met the following criteria: (1) diagnosis of SZ according to the Diagnostic and Statistical Manual of Mental Disorders, Fifth Edition (DSM-5) by two independent experienced psychiatrists, and (2) aged 18–44 years old. Patients were excluded if they: 1) had comorbid severe physical diseases, cardiovascular diseases, diabetes, hypertension, other metabolic or endocrine diseases, infectious diseases, or immune system diseases; 2) comorbid severe neurological diseases or intellectual disability; 3) were pregnant or lactating; 4) had an intellectual disability; 5) had substance (drug and alcohol) abuse; 6) significant fluctuations in psychotic symptoms in the past two weeks. Of the 301 patients, 125 were treated with a single antipsychotic and 176 with a combination of antipsychotics, including three with additional antidepressants (No Suicide Ideation group (n = 1); Suicide Ideation group (n = 2)). The daily dose of antipsychotics was converted to equivalent chlorpromazine dose for each patient.

The protocol for this study was approved by the Medical Ethics Committee of the Affiliated Kangning Hospital of Wenzhou Medical University. All participants provided written informed consent prior to formal participation. This study was conducted in strict accordance with the relevant national and international regulations.

### Measures

#### Socio-demographic characteristics

The socio-demographic data included general data (age, gender), education in years, age at first onset of psychiatric symptoms, Body Mass Index (BMI), suicide attempts (SAs), family history of schizophrenia, and antipsychotic use. Well-trained research staff collected these data via detailed surveys completed with each participant.

The first five items of the Beck Scale for Suicide Ideation—Chinese Version (BSI—CV) were used to evaluate suicidal ideation in this study. The presence or absence of suicidal ideation in patients with SZ was assessed according to the scores on items 4 and 5 of the BSI—CV. Patients were considered to be free of suicidal ideation only when they received a score of 1 for both items 4 and 5 [40]. The intensity of individual suicidal ideation was assessed based on items 1–5; higher scores (scores ranged from 0–2) reflect higher suicidal ideation intensity The BSI—CV has been shown to have good reliability and validity and has been extensively used to predict future suicide attempts and death by suicide [41].

#### Neurocognitive assessments

The Repeatable Battery for the Assessment of Neuropsychological Status (RBANS) [42] was used to assess the neurocognitive function of each participant. The scores for each test and the total RBANS score were recorded. The RBANS is made up of 12 subtests that are used to calculate five domains of cognition: (1) immediate memory: list learning and story memory; (2) visuospatial/constructional ability: figure copy and line orientation; (3) language: picture naming and semantic fluency; (4) attention: forward digit span and coding; and (5) delayed memory: list recall, list recognition, story recall, and figure recall. The subtest scores are combined to obtain a Total Score, a summary measure of RBANS performance. Reports indicate that the five-factor model is a better fit for the RBANS than the two- and three-factor alternatives [43].

#### Empathy

The IRI [44] was used to assess patients' empathy. The four-factor empathy model, as assessed by this measure, is increasingly prominent [45]. The IRI comprises 28 items and can be divided into four relatively independent subscales: 1) Perspective Taking (PT): the tendency to take a point of view and think from the standpoint of others; 2) Fantasy (FS): the ability to imagine and experience the emotions and behavior of characters in virtual environments such as books, movies, and dramas; 3) Empathic Concern (EC): feelings of warmth, sympathy, and concern for unfortunate people; a kind of "other-oriented" empathy; 4) Personal Distress (PD): feelings of anxiety and unease in a stressful interpersonal environment; a kind of "self-oriented" empathy. All items are scored on a five-point scale ranging from 0–4, reflecting "strongly disagree", "disagree", "neither agree nor disagree", "agree", and "strongly agree". A higher IRI score indicates a stronger empathy response. The IRI has been extensively used for patients with SZ and demonstrates good reliability and validity [33]. Research supports the use of the original four-factor structure of the scale for empathy assessments rather than the two-factor alternative [45].

#### Clinical assessments

The psychopathological symptoms of the patients with SZ were assessed by two independent psychiatrists using the Positive and Negative Syndrome Scale (PANSS). This scale contains 30 items that are divided into three subscales: Positive Symptoms, Negative Symptoms, and General Psychopathology, The higher the score, the more severe the symptoms [46].

### Statistical analyses

Comparisons between the patients with suicidal ideation and those without suicidal ideation were made using t-tests and the χ2 test for continuous and categorical variables, respectively. The false discovery rate (FDR) correction was used to adjust the t-tests for multiple comparisons. Then, when appropriate, Pearson and Spearman correlations were performed (neurocognitive functions, empathy, and clinical symptoms). Furthermore, binary logistic stepwise regression analysis was used to analyze the relationship between suicidal ideation and the factors that showed significant differences between groups in the results of the previous statistical analysis(i.e., Age, Gender, Suicide attempts, PANSS-positive symptoms, PANSS-Negative symptoms, PANSS-general psychopathology symptoms, RBANS- Language and IRI- Personal Distress score; the PANSS Total Score was not included in the regression model given that the PANSS Total Score includes the Positive Symptoms and General Psychopathology scores) in order to identify the factors most strongly associated with suicidal ideation among young patients with SZ in China. Finally, a plug-in program was used in SPSS to perform moderation and mediation effect testing. All statistical analyses were performed using IBM SPSS Statistics 26. The threshold for statistical significance was *P* < 0.05.

## Results

In total, 301 patients with SZ spectrum disorders participated in this study. The mean age of the participants was 33.93 (SD = 6.37) years. The sample comprised 183 men (60.8%) and 62 patients with a family history of schizophrenia (20.6%). Moreover, 82 patients (27.2%) reported suicidal ideation in the last week or when they were most depressed.

### Sociodemographic characteristics

The sociodemographic characteristics of the patients with and without suicidal ideation are presented in Table 1. Suicide attempts and BSI-CV-suicide ideation significantly differed among the two groups (all FDR-corrected* P* < 0.001).

**Table 1:** Demographic characteristics of patients with or without suicide ideation, N (%) or M (SD)

Variables	No Suicide Ideation (*N* = 219)	Suicide Ideation (*N* = 82)	t/χ^2^	*P*
Age (year)	33.71(6.42)	34.52(6.25)	-0.990	0.323
Education years	9.58(3.02)	9.63(3.87)	-0.105	0.917
Age at first onset of psychiatric symptoms	22.76(5.74)	23.06(5.97)	-0.403	0.687
BMI	25.08(4.84)	24.48(4.26)	0.993	0.321
Suicide attempts	0.10(0.37)	0.56(0.80)	-6.894	0.000***
Gender (Male/ Female)	136/83	47/35	0.573	0.449
Family history (no/yes)	178/41	61/21	1.731	0.188
Antipsychotics (single/combination)	96/123	29/53	1.763	0.184
Typical antipsychotics dose (CPZ equivalent mg)	27.68 (126.99)	34.91(134.97)	-0.432	0.666
Atypical antipsychotics dose (CPZ equivalent mg)	462.97(286.60)	431.49(251.72)	0.876	0.382
Antipsychotics total dose (CPZ equivalent mg)	489.15(292.27)	462.40(264.66)	0.725	0.469
BSI-CV-suicide ideation	0.36(0.99)	7.55(3.90)	-25.241	0.000***

*M* Mean, *SD* Standard deviation, *BMI* Body mass index, *CPZ* Chlorpromazine, *BSI-CV* Beck Scale for Suicide Ideation—Chinese Version. ***significant at *P* < 0 .001

### Suicidal ideation and associations

The RBANS, IRI, and PANSS scores for the patients with and without suicidal ideation are presented in Table 2. A total of 301 patients (including 82 with and 219 without suicidal ideation) completed the questionnaire. After FDR correction, patients with and without suicidal ideation did not show significant differences in performance on the RBANS-Language score (mean = 77.84 (13.64) vs. mean = 81.69 (15.37); *P* = 0.047; FDR-corrected *P* = 0.141 > 0.05). Further, there was a statistically significant difference in IRI-Personal Distress between the patients with suicidal ideation and those without suicidal ideation. Patients with suicidal ideation had a higher average IRI-Personal Distress subscale score (mean = 22.46 (5.19) vs. mean = 20.47 (4.72); *P* = 0.002; FDR-corrected *P* = 0.010 < 0.05) than those without suicidal ideation. Patients with suicidal ideation also had a significantly higher PANSS-Total score (mean = 82.44 (17.42) vs. mean = 75.09 (16.47); *P* = 0.001; FDR-corrected *P* = 0.008 < 0.05). PANSS-Positive Symptom score (18.55 (6.31) vs 16.39 (5.57); *P* = 0.004; FDR-corrected *P* = 0.015 < 0.05), and PANSS-General Psychopathology score (42.72 (8.28) vs 38.23 (8.32); *P* < 0.001; FDR-corrected *P* < 0.001) compared to patients without suicidal ideation.

**Table 2:** Neurocognitive functions, empathy and Clinical variables scores between patients with or without suicide ideation

Variables	No Suicide Ideation (*N* = 219)	Suicide Ideation (*N* = 82)	t	*P*
**RBANS scores**				
Total	67.55(13.96)	64.89(12.73)	1.507	0.133
Immediate memory	59.60(16.80)	64.91(57.15)	-1.242	0.215
Visuospatial	77.58(17.71)	75.27(17.36)	1.014	0.312
Language	81.69(15.37)	77.84(13.64)	1.994	0.047*
Attention	81.74(16.30)	78.39(16.10)	1.595	0.112
Delayed memory	67.52(19.75)	65.98(19.67)	0.603	0.547
**IRI scores**				
Total	85.80(13.97)	86.13(13.85)	-0.186	0.853
Perspective-Taking (PT)	22.37(5.06)	21.27(4.92)	1.701	0.090
Fantasy (FS)	20.58(5.32)	20.15(5.90)	0.610	0.542
Empathic Concern (EC)	22.38(4.94)	22.26(5.12)	0.190	0.849
Personal Distress (PD)	20.47(4.72)	22.46(5.19)	-3.166	0.002**
**PANSS scores**				
Total	75.09(16.47)	82.44(17.42)	-3.394	0.001**
Positive symptoms	16.39(5.57)	18.55(6.31)	-2.882	0.004**
Negative symptoms	20.46(6.33)	21.17(6.89)	-0.845	0.399
General psychopathology	38.23(8.32)	42.72(8.28)	-4.170	0.000***

*M* Mean, *SD* Standard deviation, *RBANS* Repeatable Battery for the Assessment of Neuropsychological Status, *IRI* Interpersonal Reactivity Index, *PANSS* Positive and Negative Syndrome Scale. *Significant at *P* < 0 .05; **significant at *P* < 0 .01; ***significant at *P* < 0 .001

### Relationships between neurocognitive functions, empathy, and clinical symptoms

The correlation coefficients between neurocognitive functions and empathy for all patients are shown in Table 3. The IRI Total Score and its subscales (PT, FS, and EC) were positively associated with the RBANS Total Score and its subscales (Immediate memory, Language, and Delayed memory) (all *P* < 0.05).

**Table 3:** Pearson correlation coefficients for neurocognitive functions and empathy

	IRI-PT	IRI-FS	IRI-EC	IRI-PD	IRI Total scores
RBANS-Immediate memory	.114^*^	-0.030	.116^*^	0.059	0.092
RBANS-Visuospatial	0.049	-0.025	0.001	-0.032	-0.004
RBANS-Language	.114^*^	.161^**^	0.104	0.002	.142^*^
RBANS-Attention	0.086	0.087	-0.012	-0.020	0.054
RBANS-Delayed memory	.159^**^	-0.004	0.043	-0.047	0.054
RBANS Total scores	.134^*^	0.063	0.054	-0.019	0.085

*RBANS* Repeatable Battery for the Assessment of Neuropsychological Status, *IRI* Interpersonal Reactivity Index, *PT* Perspective Taking, *FS* Fantasy, *EC* Empathic Concern, *PD* Personal Distress. *Significant at *P* < 0 .05; **significant at *P* < 0 .01

The correlation coefficients between neurocognitive functions, empathy, and clinical symptoms for the No SI and With SI subgroups are shown in Table 4. Females exhibited poorer performance on the RBANS Total Score and its subscales (Immediate Memory, Visuospatial, Language, Attention). The PANSS Positive Symptoms, Negative Symptoms, General Psychopathology, and Total Score were negatively associated with the RBANS Total Score and its subscales (all *P* < 0.05). In the No SI group, the IRI Total Score and its subscales (PT and EC) were negatively associated with the PANSS Negative Symptoms, General Psychopathology, and Total Score (all *P* < 0.05). In the With SI group, there were no significant correlations between the IRI and any other variables.

**Table 4:** Pearson correlation coefficients for neurocognitive functions, empathy and clinical symptoms

Variables	Age		Gender^a^		Positive symptoms		Negative symptoms		General psychopathology		PANSS- Total scores	
	No SI	With SI	No SI	With SI	No SI	With SI	No SI	With SI	No SI	With SI	No SI	With SI
RBANS												
Immediate memory	-0.018	0.072	-0.015	-0.126	-.138^*^	-0.185	-.263^**^	-0.176	-.324^**^	-0.197	-.311^**^	-.230^*^
Visuospatial	0.072	0.024	-.259^**^	-.424^**^	-.217^**^	-0.208	-.259^**^	-0.072	-.257^**^	-.263^*^	-.303^**^	-.229^*^
Language	0.079	-0.054	-.258^**^	-.219^*^	-0.068	-0.189	-.221^**^	-.302^**^	-.219^**^	-.266^*^	-.219^**^	-.314^**^
Attention	-0.040	-0.134	-0.078	-.305^**^	-0.107	-.273^*^	-.182^**^	-.333^**^	-.235^**^	-.357^**^	-.225^**^	-.400^**^
Delayed memory	0.031	0.037	-0.062	-0.144	-.155^*^	-0.195	-.303^**^	-.226^*^	-.350^**^	-.376^**^	-.346^**^	-.339^**^
Total scores	0.018	0.030	-.180^**^	-.312^**^	-.198^**^	-.286^**^	-.324^**^	-.297^**^	-.381^**^	-.454^**^	-.384^**^	-.436^**^
IRI												
PT	-0.011	0.038	.143^*^	-0.113	-0.078	-0.064	-.240^**^	-0.144	-.254^**^	-0.142	-.247^**^	-0.147
FS	0.074	-0.207	0.012	-0.047	0.026	0.101	-0.057	-0.105	-0.087	0.069	-0.057	0.027
EC	0.110	0.070	0.024	-0.034	-0.033	-0.001	-.153^*^	-0.054	-.180^**^	0.036	-.161^*^	-0.005
PD	0.041	-0.113	0.035	-0.125	0.031	-0.172	0.051	0.202	0.059	0.129	0.060	0.079
Total scores	0.076	-0.091	0.077	-0.119	-0.019	-0.044	-.145^*^	-0.040	-.168^*^	0.040	-.147^*^	-0.013

*No SI* No Suicide Ideation (*N* = 219), *With SI* With Suicide Ideation (*N* = 82), *RBANS* Repeatable Battery for the Assessment of Neuropsychological Status, *IRI* Interpersonal Reactivity Index, *PT* Perspective Taking, *FS* Fantasy, *EC* Empathic Concern, *PD* Personal Distress, *PANSS* Positive and Negative Syndrome Scale

^*^Significant at *P* < 0 .05; **significant at *P* < 0 .01

^a^: 1 = male; 2 = female

### Independent predictors of suicidal ideation

The binary logistic stepwise regression analysis (Table 5) revealed that Suicide attempts (OR = 4.004, 95% CI = 2.350–6.822), PANSS-General Psychopathology (OR = 1.057, 95% CI = 1.022–1.093) and IRI-PD score (OR = 1.076, 95% CI = 1.013–1.142) were significant independent predictors of suicidal ideation in SZ patients.

**Table 5:** Results of the stepwise logistic analysis: independent risk factors for schizophrenic patients with suicidal ideation

Variables	Odds Ratio (OR)	95% CI		*P*-value
		Lower	Upper	
Suicide attempts	4.004	2.350	6.822	0.000***
PANSS—General psychopathology	1.057	1.022	1.093	0.001**
IRI-Personal Distress	1.076	1.013	1.142	0.017*

Variables in the model: Age, Gender, Suicide attempts, PANSS-positive symptoms, PANSS-Negative symptoms, PANSS-general psychopathology symptoms, RBANS- Language and IRI- Personal Distress score; *PANSS* Positive and Negative Syndrome Scale, *RBANS* Repeatable Battery for the Assessment of Neuropsychological Status, *IRI* Interpersonal Reactivity Index. *Significant at *P* < 0 .05; **significant at *P* < 0 .01; ***significant at *P* < 0 .001

### Moderating and mediating effects associated with suicidal ideation

**Fig. 1:**
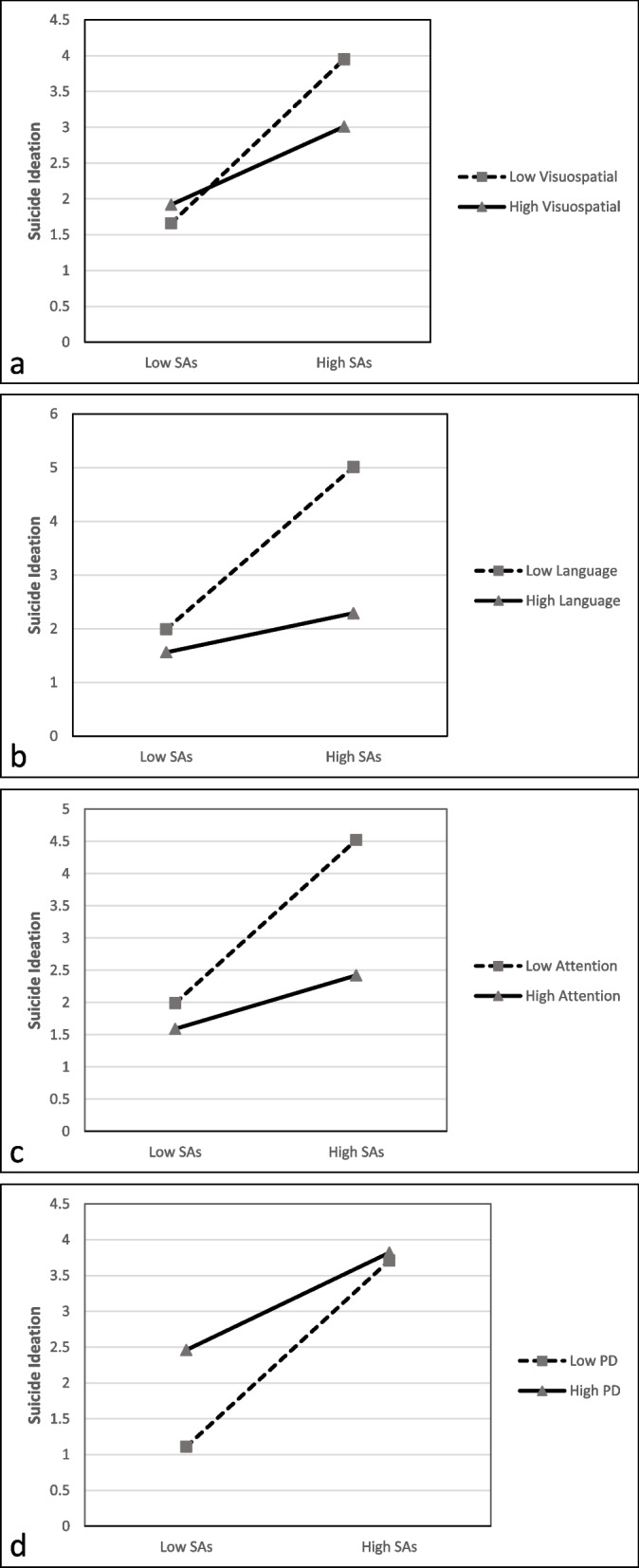
Moderation model of neurocognitive function and empathy on the relationship between suicide attempts and suicidal ideation. Note: SAs: suicide attempts; PD: Personal Distress

To illustrate the suicide attempts x neurocognitive functions and suicide attempts x empathy interactions in predicting suicidal ideation, the regression lines were plotted according to the procedures outlined in a previous study [47]. Then, the moderating effects between suicide attempts and the IRI and RBANS Total Scores and subscales were explored; the moderating effects were significant for the RBANS-Visuospatial, RBANS-Language, RBANS-Attention, and IRI-PD. As shown in Fig. 1a, b, c, and d, the relationship between suicide attempts and suicide ideation weakened as the RBANS-Visuospatial, RBANS-Language, RBANS-Attention, and IRI-PD scores increased.

**Fig. 2:**
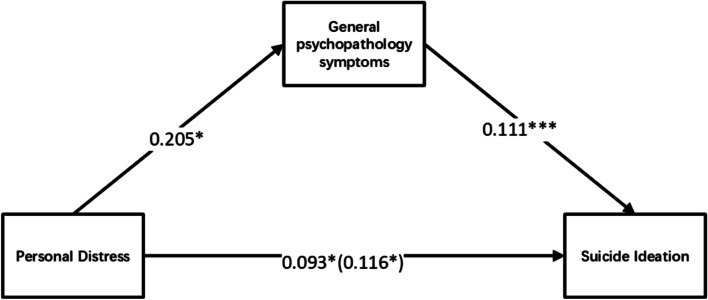
Mediation model of personal distress, general psychopathology symptoms and suicidal ideation. Note: *Significant at *P* < 0 .05; ***significant at *P* < 0 .001

Both the PANSS-General Psychopathology Symptoms and IRI-Personal Distress scores were significantly related to suicidal ideation. There is a similar concept and pattern of "anxiety and discomfort" in both the "personal distress" aspect of empathy and the "general psychopathology symptoms" of the PANSS. Therefore, a mediation model of personal distress—general psychopathology symptoms—suicidal ideation might exist. Based on the theoretical assumptions [48], a mediating effect model was constructed (suicidal ideation as the dependent variable, the IRI-Personal Distress score as the independent variable, the PANSS-General Psychopathology Symptoms score as the intermediary variable). As shown in Fig. 2, the direct effect of IRI-Personal Distress on suicidal ideation was 0.093 (*p* = 0.036, 95% CI: 0.006–0.180) and the mediating effect of General Psychopathology Symptoms on the relationship between personal distress and suicidal ideation was 0.023 (95% CI: 0.003–0.048). Further, the total effect of the personal distress—general psychopathology symptoms—suicidal ideation model was 0.116 (95% CI: 0.027–0.205). The interval of the indirect effect did not contain 0, indicating that it was statistically significant. Therefore, General Psychopathology Symptoms played a partial mediating role in the relationship between personal distress and suicidal ideation in.

## Discussion

This study attempted to combine three important topics of interest (suicidal ideation, neurocognitive function, and empathy) in Chinese adults with SZ. In this study, the prevalence of suicidal ideation (27.2%) among Chinese adults with SZ was similar to that reported among patients with SZ in China (25.8%) [49], but lower than the global set of lifetime prevalence of SI mentioned in another meta-analysis (34.5%, 95% CI: 28.2 − 40.9%) [1]. The reported rates of suicidal ideation among people with SZ vary widely. The reasons for these differences in the prevalence of suicidal ideation include different demographic characteristics of the patients, different illness stages, and various comorbidities [50]. Therefore, caution should be exercised when directly comparing the findings of the current study with those of other studies.

The current study did not find a relationship between the RBANS score and suicidal ideation among patients with SZ. This is similar to the results of previous studies [20, 21], and suggests that suicidal ideation among patients with SZ might not be related to neurocognitive function. However, the results of previous studies are inconsistent, and some studies [14–16] have reported that impaired neurocognition in patients with SZ is a risk factor for suicide while others suggest that the risk of suicide in patients with SZ increases with improved cognitive function [17–19]. Thus, given that the reported relationship between suicidal ideation and neurocognitive in patients with SZ is inconsistent, further prospective studies with larger samples are needed. The results of the current study were consistent with previous studies. Female patients with SZ were found to have lower RBANS scores than males [51], suggesting more neurocognitive impairment in females. Further, the RBANS Total Score and its subscales were negatively associated with PANSS Positive Symptoms, Negative Symptoms, General Psychopathology, and Total Score. There is one possible explanation for this negative correlation. Generally speaking, patients with SZ who take antipsychotic drugs tend to achieve remission of psychotic symptoms and a decrease in PANSS scale scores. However, cognitive deficits usually improve less with time and treatment [52, 53].

Interestingly, the results of the current study indicated that empathy, especially the IRI-Personal Distress subscale score, was higher in patients with suicidal ideation, as compared to those without suicidal ideation. Further, the results of the binary logistic stepwise regression analysis confirmed the relationship between suicidal ideation and the IRI-Personal Distress subscale. This result is consistent with a study of previous suicide attempts among Chinese patients with SZ [54], where suicide attempters were found to experience greater personal distress compared to those who did not attempt suicide. It is possible that people with SZ who score higher on personal distress have higher levels of anxiety and discomfort, which adversely impacts their interpersonal and social support, further contributing to suicidal ideation. Some studies have reported significant positive associations between personal distress subscale scores and negative affect, poor emotion regulation [55], and depressive symptoms [56], and a negative association with personal quality of life [57]. These are also risk factors for suicide [7, 58, 59]. Thus, it can be speculated that personal distress may also indirectly influence suicidal ideation through these risk factors.

In addition to psychosocial factors, neurobiological studies have found a positive correlation between IRI-Personal Distress subscale scores and blood oxygen level-dependent activities in the right temporal pole that are related to social behavior and functioning [60, 61]. Another study reported that psychiatric patients who died of suicide had a greater density of von Economo neurons in the anterior cingulate cortex, as compared to patients who died from other causes [62]. The paracingulate, anterior and posterior cingulate, and amygdala are closely related to empathy [34]. Therefore, it is possible that the positive relationship between IRI-Personal Distress and suicide ideation is related to changes in brain structures such as the anterior cingulate cortex. In addition, in the No Suicide Ideation group, the IRI-PT, EC, and Total Score were negatively correlated with the PANSS Negative Symptoms score, General Psychopathology score, and Total score. The severity of clinical symptoms may be a factor in predicting empathy in patients with SZ [63].

The results of this study also indicated that higher PANSS-General Psychopathology scores were associated with increased suicidal ideation in patients with SZ. This is consistent with previous studies which have found that general psychopathology symptoms are correlated with high suicide risk [64, 65]. Further, impulsivity is positively correlated with the PANSS-General Psychopathology score, and impulsivity is associated with suicide in patients with SZ [66]. However, some studies have not found a significant association between general psychopathology and suicide risk [67]. These varied results may be due to sample differences, such as differences in ethnicity or disease progression. Studies employing a longitudinal design with a larger sample are much needed to clarify these inconsistencies. Further, the current study found that the PANSS-General Psychopathology Symptoms score played a partial mediating role in the relationship between personal distress and suicidal ideation. This may be due to the similar concept and pattern of "anxiety and discomfort" in both the "personal distress" aspect of empathy [44] and the "general psychopathology symptoms" of the PANSS [46]. Thus, this mediating effect may be due to the inclusion of a common component. More studies are needed to verify these preliminary findings.

Additionally, high suicide attempts was found to be associated with increased suicidal ideation in people with SZ, similar to a previous study [68]. Suicide is a continuous process, and more suicide attempts may lead to more suicides [69]. Of interest, the results of the moderating effects show that this relationship weakened with increases in the RBANS-Visuospatial, Language, Attention, and IRI-PD scores. This suggests that higher neurocognitive function and empathy may effectively reduce the incidence of suicidal ideation associated with suicide attempts. The inclusion of neurocognition and empathy as moderator variables in this study provides a new perspective, with the findings suggesting that improving the RBANS-Visuospatial, Language, Attention, and IRI-PD scores of patients with SZ who have made suicide attempts is a possible means to reduce suicidal ideation. Nonetheless, this preliminary finding should be verified in future studies.

Previous cross-sectional and longitudinal studies of the relationship between neurocognition and social cognition have found that these two variables are related but distinct factors, and improving neurocognitive impairments and social cognition impairments requires different approaches [23, 70]. This study aimed to explore neurocognition and empathy as influential factors in suicidal ideation among patients with SZ. The results of this study provide relevant information about the role of personal distress within the concept of empathy and its association with suicidal ideation among patients with SZ. These findings offer a deeper understanding of the factors influencing suicidal ideation in patients with SZ, and this may inform the development of prevention and intervention approaches.

Several limitations of the present study should be noted. First, this cross-sectional study was not able to investigate the causal relationships between suicidal ideation and risk factors in Chinese adults with SZ. Thus, we cannot confirm whether there are direct causal relationships between suicide attempts and neurocognitive impairment, empathy, and clinical symptoms in patients with SZ. As such, the main findings of this study should be considered to be preliminary. Second, this study focused on patients with SZ and did not include a healthy control group. Thus, the results obtained in this study are limited. Future studies should include healthy controls as a comparison group. Third, the BSI—CV, which was used to assess suicidal ideation is a subjective scale. Nonetheless, it is widely used in clinical practice. Finally, unfortunately, family history of depression and family history of suicide were not included as variables in the current study, which may have limited the findings. These variables should be included in future studies of suicidal ideation among patients with SZ.

## Conclusions

The results of this study indicate that the personal distress aspect of empathy, the severity of general psychopathological symptoms and suicide attempts are important predictors of suicidal ideation among Chinese adults with SZ. Moreover, the relationship between suicide attempts and suicide ideation was found to weaken with increases in empathy (personal distress) and neurocognitive function (visuospatial, language, and attention). This suggests that addressing neurocognitive function and empathy may play an important role in the prevention and treatment of suicidal ideation in patients with SZ. Future prospective studies with larger sample sizes are needed to validate the relationships between neurocognitive function, empathy, and suicidal ideation in patients with SZ.

## Data Availability

The raw data supporting the conclusions of this article will be made available by the authors, without undue reservation. The datasets used and/or analysed during the current study available from the corresponding author on reasonable request.

## Abbreviations

SZ: Schizophrenia
BSI—CV: Beck Scale for Suicide Ideation—Chinese Version
RBANS: Repeatable Battery for the Assessment of Neuropsychological Status
PANSS: The Positive and Negative Syndrome Scale
IRI: Interpersonal Reactivity Index
PT: Perspective Taking
EC: Empathic Concern
PD: Personal Distress
FS: Fantasy
No SI: No Suicide Ideation
SI: Suicide Ideation
SA: Suicide attempts
